# Impact of informal caregiving on caregivers’ subjective well-being in China: a longitudinal study

**DOI:** 10.1186/s13690-023-01220-1

**Published:** 2023-12-06

**Authors:** Mingmei Cheng, Hualei Yang, Qian Yu

**Affiliations:** 1 School of Public Finance and Taxation, Zhongnan University of Economics and Law, Wuhan, China; 2 School of Public Administration, Zhongnan University of Economics and Law, Wuhan, China

**Keywords:** Informal caregiving, Subjective well-being, Population ageing, China

## Abstract

**Background:**

While informal caregiving is crucial for improving and maintaining health of the elderly, there is limited evidence of its potential effect on caregivers’ wellbeing. Understanding this effect is important for policy makers to design effective long-term care policies. This longitudinal study aims to investigate the impact of informal caregiving on caregivers’ subjective wellbeing in China.

**Methods:**

Three waves (2016, 2018, 2020) of data from the China Family Panel Studies (CFPS) are constructed for empirical analysis. Ordered logit model is first used to estimate the effect. Fixed effects ordered logit model and mixed effects ordered logit model are further employed to control for the possible bias from unobserved individual heterogeneity.

**Results:**

Informal caregiving significantly reduces caregivers’ subjective wellbeing and the negative effect is stronger for high-frequency caregivers. Subgroup analysis reveals that informal caregiving imposed greater negative impacts on women, those living in rural areas, being married, working, and living separately from parents. Further analysis of mechanism indicates that decrease in wage income, leisure and sleep time were channels through which informal caregiving affects caregivers’ well-being.

**Conclusion:**

When policy makers formulate sustainable long-term care policies and home support services, interventions to improve caregivers’ stress-coping skills and ensure their engagement in leisure and social activities could be adopted to mitigate the negative effects on caregivers’ subjective well-being.

**Table:** 

Text box 1. Contributions to literature
• This study adds to the literature by examining the impact of informal caregiving on the caregivers’ subjective wellbeing in China
• Informal caregiving significantly reduces caregivers’ subjective wellbeing and the negative effect is stronger for high-frequency caregivers
• Informal caregiving had greater adverse effects on women, those living in rural areas, being married, working, living separately from parents, and having children
• Increase in depression symptoms and decrease in wage income, leisure and sleep time are channels through which informal caregiving affects caregivers’ wellbeing

## Introduction

Increasing life expectancy, combined with declining fertility rate, has accelerated population ageing in China. By the end of 2020, the number of people over 60 years old in China reached 264 million, accounting for 18.7% of the total population [1]. The proportion aged 60 and above in China is expected to reach 32.8% by 2050 [2]. At the same time, the proportion of disabled elderly aged 65 and above in China is predicted to be 13.68% in 2050 [3]. In concordance with the rapid socio-demographic transition, the demand for elderly care is rising drastically. The ratio of old persons in need of care is 15.2% for those with normal functions, 78.4% for the partially disabled and 96.7% for the totally disabled [4]. Accordingly, the cost of providing care for older adults with moderately impaired ADLs will increase from $898 million in 2020 to $3,928 million by 2050 in China [5]. Thus, it becomes a serious concern for policy makers on how to implement the sustainable long-term care policies and support services in China.

At present, there are three modes of providing care for older people in China, which include home-based informal caregiving, community-based residential care, and institutionalized care. Informal care is usually defined as assistance and support provided by a spouse, children, or other family members to older persons with no paid compensation [6]. Owing to the influence of filial piety of Confucius philosophy and infancy of long-term care insurance in China, most of the elderly care is undertaken by family members [7]. The estimated economic value of informal care is over $58.72 billion per year [8]. Despite the great value of informal care, its potential cost for caregivers has received relatively little attention in previous studies.

Theoretically, the impact of informal caregiving on caregivers is complex, with both positive and negative effects. On the one hand, informal caregiving enhances the affinity between caregivers and older adults through positive feedback from the caregiving process, such as emotional satisfaction and cognitive growth experience [9]. Caregivers can get satisfaction from caregiving process [10]. Moreover, caregivers also provide informal care out of altruism and volunteerism, which are potential sources of communication and social engagement [11, 12]. On the other hand, caregiving is time-consuming, mentally stressful, and physically demanding [13]. It leads to loss of employment opportunities, reduction in working time and wages, and fewer social activities, which may decrease caregivers’ well-being [14, 15]. Therefore, it becomes an empirical question to determine which effect dominates.

Although a few studies have investigated the well-being of informal caregivers, their findings are mixed [16, 17]. On the negative effect, Van den Berg et al. find that providing home elderly care reduces caregivers’ subjective well-being [18]. Van den Berg et al. suggest that a significant negative relationship between informal caregiving and caregivers’ subjective well-being [15]. However, those studies were limited to a cross-sectional sample recruited from care support centers and may suffer from selectivity bias. By contrast, several studies documented that informal caregiving has positive effects on caregivers’ well-being. Cohen et al. report that 80% of caregivers in Canada reported positive feelings towards caregiving, in which companionship and a fulfilling or rewarding feeling occur most [19]. Chappell and Reid argue that caregivers’ overall quality of life can be improved even if they experience caregiving burden since social support is strongly related to well-being [20]. Trukeschitz et al. report that caregivers may experience competence, mastery, and self-esteem in their caregiving role [21]. Based on 4,817 adults from the 2014/15 UK Time Use survey, Urwin et al. find that informal caregivers have higher level of experienced well-being than non-carers do [22].

At present, little is known about how the effect works in the Chinese settings. Chen et al. is the first study to use China Health and Nutrition Survey (CHNS) and show that informal care significantly reduced the subjective wellbeing of female caregivers [23]. Nevertheless, this study used the data collected more than ten years ago (2009 and 2011 waves of CHNS) and cannot reflect the latest development of informal caregiving in China. Liu et al. employ the sample from 310 caregivers in Shanghai and report that providing informal care decrease self-reported wellbeing of the caregivers [24]. However, this study suffers from two limitations. First, it is silent on the causal effect of informal care on caregivers’ subjective wellbeing. Second, the findings cannot be generalized to general population because it only focuses on the institutions’ caregivers in Shanghai.

Using three waves (2016, 2018, 2020) of data from the China Family Panel Studies (CFPS), we empirically investigate the effect informal caregiving on caregivers’ subjective well-being. We employ fixed effects ordered logit model to control for the potential bias from unobserved individual heterogeneity. We find strong evidence of a negative effect of informal caregiving on caregivers’ subjective well-being, and the effect was greater for those who provided high frequency of care. The results remain stable after a series of robust checks. Our results also reveal that informal caregiving has greater negative impacts on women, those living in rural areas, being married, working, and living separately from parents. Further mechanism analysis indicates that informal caregiving negatively affected caregivers’ well-being mainly through decrease in wage income, leisure, and sleep time.

Our study contributes to the literature in three aspects. First, although most previous studies examined the effects of caregiving in western countries, our study enriched the literature by providing the latest evidence from China. Second, our study complements the research on Socioeconomic Status-wellbeing relationship by exploring the heterogeneous effects of informal caregiving on caregiver’s well-being by the socio-demographic characteristics. Third, most previous studies have been silent on the channels behind the effects. In contrast, we explore the three possible channels underlying the effects.

## Stress process model

In this study, the stress process model provides helpful insights for understanding how informal caregiving affects caregiver’s wellbeing [25]. According to the stress process model, informal caregiving is a source of stress and has a detrimental effect on caregivers’ wellbeing [26]. Moreover, the extent to which caregivers experience care-giving as burdensome or stressful is influenced by a variety of background characteristics of the caregiver such as age, gender, and socioeconomic status, which define the social and personal resources available to cope with the challenges of caregiving [27]. As such, informal caregiving is likely to have heterogenous effects on caregiver’s wellbeing.

There are three possible mechanisms through which informal caregiving affects caregivers’ wellbeing. First, as a potential stressful event, informal caregiving is detrimental to caregivers’ wellbeing if caregiving demands are beyond caregivers’ psychological and social resources [28]. Previous studies find that compared with non-caregivers, caregivers generally suffer from greater psychological stress and are more likely to have psychological stress responses such as depression, anxiety, and irritability. Bassoli et al. suggest that caring for a parent close to death could lead to depression, especially for daughters [29]. Caregiving also aggravates the diseases of caregivers, such as insomnia, chest pain [30]. Second, as a time-intensive domestic task, the provision of informal caregiving means less time for labor market and leisure activities. Existing research suggests that leisure time can enhance individuals’ subjective well-being by improving social support. Thus, caregiving makes caregivers more isolated and disconnected [31]. Third, informal caregiving can increase the financial burden of caregivers. Informal caregiving significantly also reduces labor force participation and imposes explicit economic costs on caregivers, such as cash expenditures for medical care and daily necessities involved in providing care [32].

Based on stress process model and previous research findings, the following hypothesis are examined in this study:

### Hypothesis 1

Informal care has adverse effects on caregiver’s subjective wellbeing.

### Hypothesis 2

The negative effect is stronger for people with lower socioeconomic status, i.e., women, unmarried, those living in rural areas and with low income.

### Hypothesis 3

Decrease in wage income and reduction in leisure and social activities are the possible channels through with informal caregiving affects caregivers’ subjective wellbeing.

## Research design

### Data

**Fig. 1:**
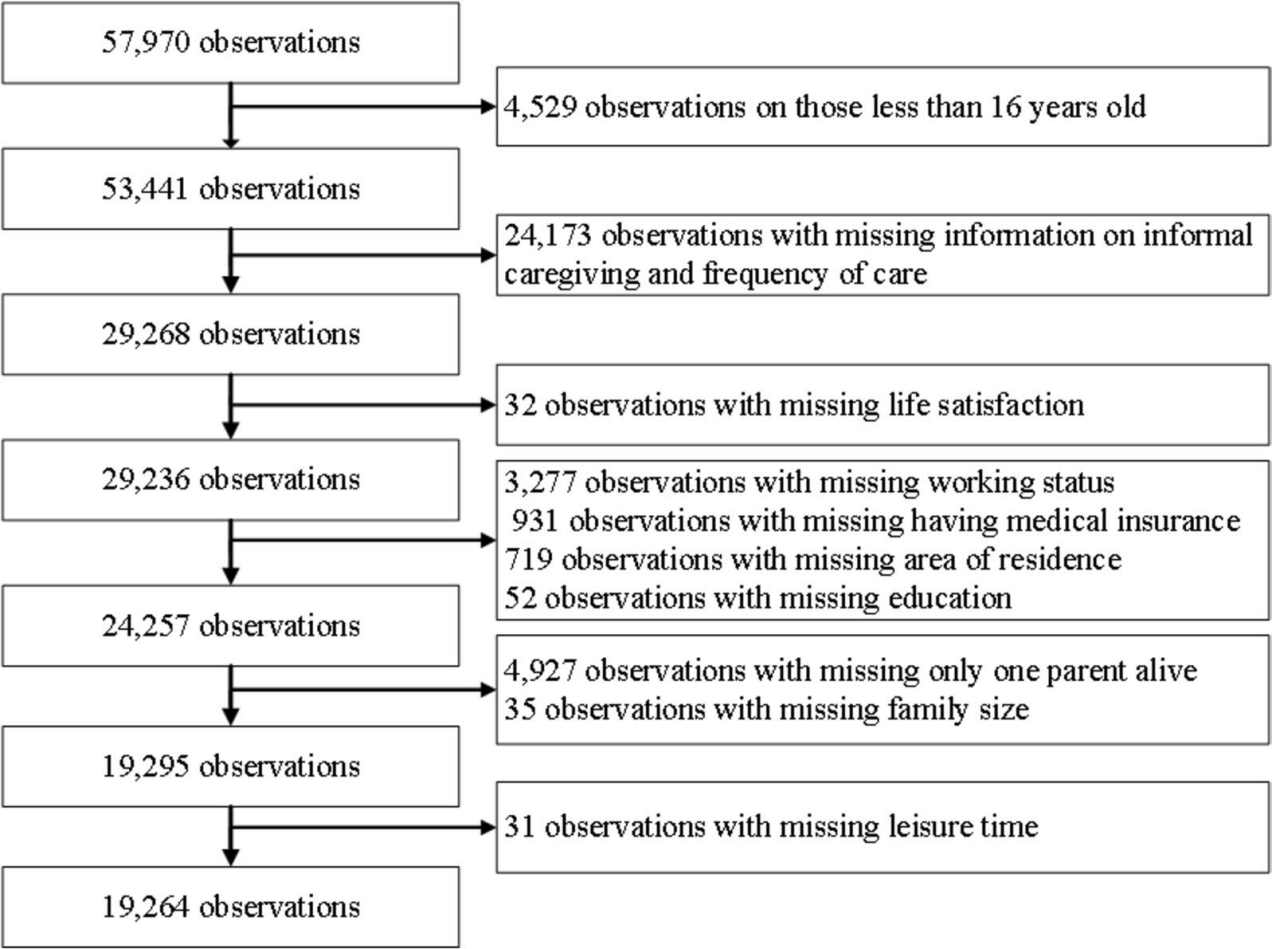
Flow chart on how the final analytical sample was derived from the China family panel studies 2016, 2018,2020

Our data is drawn from the waves of 2016, 2018 and 2020 of the China Family Panel Studies (CFPS). The CFPS is a nationally representative, large-scale longitudinal survey which was implemented by the Institute of Social Science Survey (ISSS) of Peking University. The CFPS covers 25 provinces (municipalities and autonomous regions) in China, and surveys all family members in the sampled households. It is designed to collect detailed information on sociodemographic characteristics, health status, chronic diseases, family and social relationships and health behaviors. For this study, we focus on adults aged 16 and above. After deleting observations with missing values, the final sample consists of 19,264 observations. Figure 1 provides a flow chart illustrating how the final analytical sample was derived.

### Outcome variable: subjective well-being

Life satisfaction was used to assess caregivers’ subjective wellbeing. Life satisfaction is the commonly used subjective well-being measure and often used and recommended as a suitable overall summary indicator of subjective well-being [33]. In CFPS, respondents were asked: “How satisfied are you with your life?”. There were five responses: “very dissatisfied”, “dissatisfied”, “fair”, “satisfied”, “very satisfied”, and “very satisfied”, which were assigned on an ordinal scale from 1 (very dissatisfied) to 5 (very satisfied).

### Exposure variable: informal caregiving

We used two variables to measure informal caregiving: whether respondents provided informal caregiving and frequency of care. For the first variable, the CFPS asked respondents: *“Over the past 6 months, did you take care of your father’s/mother’s household chores or his/her meals?*” A binary indicator was given a value of 1 if respondent answered “yes” and 0 if the respondent answered “no.” The second variable was created from the question: *“Over the past 6 months, how often did you perform household chores for your father/mother or take care of his/her food and living?”* The frequency of care was defined as high frequency of care if the response is *“almost every day”* and as low frequency of care if the response is *“3–4 times a week”, “1–2 times a week”, “2–3 times a month”* and *“one time a month”*.

### Covariates

Based on previous studies [23, 34], we control for a set of socioeconomic, demographic and household characteristics which include age, gender(1 = female, 0 = male), marital status(1 = married, 0 = others), area of residence(1 = urban, 0 = rural), having medical insurance(1 = yes, 0 = no), the education variable included three categories: primary school or less, secondary school, and college or more; working status(1 = working, 0 = not working), log of income per capita, having chronic diseases (1 = yes, 0 = no), smoking(1 = yes, 0 = no), drinking(1 = yes, 0 = no), whether they lived with parents(1 = yes, 0 = no), whether they have children(1 = yes, 0 = no), whether one of the parents is still alive (yes = 1,0 = no) and family size.

### Mechanism variables

We select four variables to examine the channels through which informal caregiving affects caregivers’ subjective wellbeing. First, we employed the depression index developed by Centre for Epidemiological Studies Depression Scale (CES-D) to assess caregivers’ psychological stress. It is constructed from eight questions about respondents’ feelings and perceptions over the past week, such as what extent they felt depressed, everything was an effort, poor quality of sleep, hopeful, lonely, happy, had trouble keeping mind, and could not get “going”. All responses are rated on a three-point scale ranging from 0 (almost none) to 3 (most of all the time). The scores for the eight questions are then totaled and the sum score ranged from 0 to 24, with a higher score indicating more depressive symptoms. Second, we used the logarithm of wage income to measure the effect of informal care on caregivers’ loss of job opportunities. Third, we further used leisure time and sleep time to measure the time cost of caregiving.

### Econometric model

Given that subjective wellbeing is measured on categorical scale, ordinary least squares (OLS) may not be appropriate in this case. To account for the nonlinear nature of our dependent variable, we employed the ordered logit model to estimate the impact of informal caregiving on caregivers’ subjective wellbeing, which can be specified as follows:

1 $${W}_{it}^{*}=\beta Car{e}_{it}+{X}_{it}\delta +{\gamma }_{i}+{\lambda }_{t}+{\epsilon }_{it}, t=1,\cdots ,3;i=1,\cdots ,N.$$

2 $${W}_{it}=k if {\tau }_{ik}<{W}_{it}^{*}\le {\tau }_{k+1} k=1,\cdots ,5$$

Where,${W}_{it }^{*}$ denotes the latent variable of caregivers’ subjective wellbeing, ${W}_{it}$ refers to the observed subjective wellbeing of the caregivers. ${Care}_{it }$refers to informal caregiving provided by individual $i$at time $t$. ${X}_{it }$represents a set of socioeconomic, demographic, and household variables, ${\gamma }_{i }$is unobservable individual fixed effects and ${\lambda }_{t}$ is year fixed effects. ${\alpha }_{0 }$ is an intercept term, and ${\epsilon }_{it }$ is the random disturbance term. ${\tau }_{ik}$are the response thresholds which are assumed to be strictly increasing (${\tau }_{k}<{\tau }_{k+1}$ ∀k) and${\tau }_{0}$=$-{\infty }$, ${\tau }_{k+1}$=${\infty }$, and $k$is the response categories for wellbeing, taking values from 1 to 5. $\beta$is the coefficient of interest.

If the error term in Eq. (1) is uncorrelated to informal caregiving decision ordered logit model will yield unbiased and consistent estimator. However, informal caregiving decisions may be endogenous. There are some unobservable factors, such as family circumstances and work preferences, which may cause informal caregiving to be related to the error term systematically. Moreover, caregiver’s well-being status may also affect his/her informal caregiving decision. Individuals with subjective well-being are more likely to provide informal caregiving. To address the endogeneity of informal caregiving, we employ fixed-effects model to control for the potential bias.

A common approach is to use fixed effects in linear regression. However, we cannot easily use linear regression procedures for fixed effects in nonlinear panels because the reliance on linear models for the analysis of categorical data can lead to inconsistent and biased effect estimates [35]. To address this issue, we apply the ‘‘blow-up-and-cluster’’ (BUC) estimator developed by Baetschmann et al.^1^ [36, 37], which collapsed the observed outcomes ${y}_{it}$ into a set of K binary variables ${d}_{it}^{k}$ with ${d}_{it}^{k}=1$ if ${y}_{it}>k$ and then using conditional maximum likelihood estimations for binary outcomes and clustering standard errors on the individual level.

We further employ mixed effects ordered logit model to estimate the impact of informal caregiving on caregivers’ subjective well-being. This model combines fixed effects, which capture relationships at the population level, with random effects, which account for variations within different clusters or groups. Therefore, it provides more accurate estimates by considering both within-subject and between-subject variability. All models are estimated in Stata 18.

## Results

### Characteristics of the study sample

Table 1 presents the characteristics of the study sample. 44.2% of the respondents provide care for their parents, and 15.8% of them provide care for their parents almost every day. Compared to non-caregivers, caregivers tend to be older, married, and more likely to live in rural areas, have health insurance, be working and have lower income and education levels. As for health and behaviors, caregivers are significantly more likely to report higher probability of chronic disease, smoking, and alcohol consumption than non-caregivers. Regarding family characteristics, compared with noncarers, caregivers are more likely to live with their parents, have slightly larger families, and have a higher proportion of only one surviving parent. In terms of mechanism variables, the wage income of caregivers is significantly lower than that of non-caregivers. They have slightly more leisure time, but relatively less sleep.

**Table 1:** Characteristics of the study sample in the China family panel studies 2016, 2018,2020

Variables	Total		Caregivers		Non-caregivers		T-test
	Mean	Std.dev.	Mean	Std.dev.	Mean	Std.dev.	
Dependent variable							
Subjective Wellbeing	3.878	0.950	3.920	0.945	3.845	0.953	***
Care variables							
Informal caregiving	0.442	0.496	1	0	0	0	
Frequency of care							
-No care provided (Ref)	0.558	0.496	0	0	1	0	
-Low-frequency of care	0.284	0.450	0.642	0.479	0	0	
-High-frequency of care	0.158	0.364	0.358	0.479	0	0	
Demographic and socioeconomic variables							
Age (16–59)	37.576	10.964	38.765	11.167	36.637	10.707	***
Female	0.432	0.495	0.411	0.492	0.449	0.497	***
Married	0.748	0.433	0.750	0.432	0.746	0.435	
Urban	0.526	0.499	0.498	0.500	0.549	0.497	***
Having Medical insurance	0.896	0.304	0.906	0.290	0.888	0.314	***
Education							
-Primary or less (Ref.)	0.280	0.448	0.292	0.454	0.270	0.443	***
-Secondary school	0.500	0.500	0.507	0.499	0.494	0.499	*
-College or more	0.220	0414	0.201	0.400	0.236	0.424	***
Working status	0.868	0.338	0.874	0.331	0.863	0.343	**
Log of income per capita	8.589	3.189	8.416	3.217	8.726	3.160	***
Having Chronic diseases	0.097	0.296	0.107	0.309	0.089	0.285	***
Smoking	0.334	0.471	0.362	0.480	0.312	0.463	***
Drinking	0.147	0.354	0.158	0.364	0.138	0.345	***
Household variables							
Living with parents	0.958	0.198	0.968	0.174	0.951	0.215	***
Having children	0.000	0.020	0.000	0.021	0.000	0.019	
Household size	4.237	2.103	4.466	2.009	4.056	2.156	***
One of the parents still alive	0.268	0.442	0.329	0.469	0.219	0.413	***
Mechanism variables							
Depression	5.521	3.812	5.545	3.779	5.502	3.839	
Log of wage income	5.331	5.342	4.993	5.296	5.598	5.364	
Leisure time	8.900	9.279	8.947	9.169	8.863	9.364	
Sleep time	7.770	1.301	7.758	1.300	7.779	1.301	***
Number of observations	19,264		8,503		10,761		

Note: ^***^ *p* < 0.01, ^**^ *p* < 0.05 ,^*^ *p* < 0.1

### The impact of informal caregiving on caregivers’ subjective well-being

Table 2 shows the effects of informal caregiving on caregivers’ subjective well-being based on ordered logit model, fixed effects ologit model and mixed effect ologit model respectively. We find strong evidence of negative impact of informal caregiving on subjective wellbeing. It can be seen from Column 1 that, compared with non-caregivers, informal caregiving significantly reduced caregiver’s probability of reporting higher wellbeing by 11%. And from Column 2, the negative impact of informal caregiving on caregivers’ subjective wellbeing still existed significantly after controlling for the unobservable fixed effects. In Column 3, the estimation from mixed effects ologit produces similar results.

**Table 2:** The impact of informal caregiving on caregivers’ subjective well-being in the China family panel studies 2016, 2018,2020

Variables	Subjective wellbeing		
	Ordered logit	Fixed effects Ologit	Mixed effects Ologit
Informal caregiving	0.887^***^ (0.027)	0.853^**^ (0.070)	0.870^***^ (0.0270)
Age	0.994^***^ (0.001)	0.906^**^ (0.045)	0.993^***^ (0.001)
Female	1.006 (0.033)	N.A.	1.047 (0.034)
Married	1.545^***^ (0.037)	1.905^***^ (0.336)	1.582^***^ (0.037)
Urban	0.957 (0.028)	0.954 (0.189)	0.931 (0.028)
Medical insurance	1.094^**^ (0.050)	1.186 (0.152)	1.117^***^ (0.045)
Secondary school	0.732^***^ (0.025)	0.643 (0.212)	0.739^***^ (0.033)
College and above	0.734^***^ (0.045)	0.416 (0.376)	0.794^***^ (0.043)
Working status	0.998 (0.041)	0.978 (0.136)	0.994 (0.046)
Log of income per capita	1.005 (0.004)	1.004 (0.012)	1.005 (0.004)
Chronic diseases	0.518^***^ (0.046)	0.742^***^ (0.093)	0.519^***^ (0.046)
Smoking	0.827^***^ (0.035)	0.660^***^ (0.105)	0.843^***^ (0.035)
Drinking	0.979 (0.071)	0.861 (0.110)	0.958 (0.069)
Living with parents	0.989 (0.072)	0.978 (0.227)	0.958 (0.069)
Having children	0.517 (0.715)	0.827 (0.961)	0.613 (0.439)
Household size	1.043^***^ (0.007)	1.074^**^ (0.037)	1.029^***^ (0.007)
One of the parents still alive	0.965 (0.033)	0.883 (0.119)	0.951 (0.032)
Pseudo R^2^	0.021	0.029	-
Observations	19,264	19,264	19,264

Note: *** *p* < 0.01, ** *p* < 0.05, * *p* < 0.1. Odds Ratio are reported and robust standard errors are in brackets. These models controlled for the dummy variables of year and province

### The impact of frequency of informal caregiving on caregivers’ subjective well-being

Table 3 provides the effect of frequency of care on subjective well-being of caregivers. As expected, coefficients of both low-frequency of care and high-frequency of care were significantly negative, suggesting that either low-frequency or high-frequency care significantly reduced caregivers’ subjective wellbeing. Moreover, high-frequency caregivers suffer larger loss of wellbeing than low-frequency caregivers.

**Table 3:** The impact of frequency of informal caregiving on caregivers’ subjective well-being in the China family panel studies 2016, 2018,2020

Variables	Subjective wellbeing		
	Ordered logit	Fixed effects Ologit	Mixed effects Ologit
Low-frequency of care	0.940^*^ (0.030)	0.974^**^ (0.012)	0.954^**^ (0.023)
High-frequency of care	0.769^***^ (0.038)	0.806^**^ (0.088)	0.782^***^ (0.031)
Age	0.994^***^ (0.001)	0.906 (0.104)	0.995^***^ (0.001)
Female	0.958 (0.033)	N.A.	0.963 (0.034)
Married	1.734^***^ (0.066)	1.906^***^ (0.336)	1.592^***^ (0.037)
Urban	0.953 (0.028)	1.042 (0.189)	0.926 (0.028)
Medical insurance	1.094^**^ (0.050)	1.190 (0.152)	1.118^**^ (0.045)
Secondary school	0.730^***^ (0.025)	0.641 (0.211)	0.737^***^ (0.033)
College and above	0.756^***^ (0.034)	0.417 (0.470)	0.790^***^ (0.043)
Working status	1.000 (0.041)	1.022 (0.137)	1.004 (0.040)
Log of income per capita	1.006 (0.004)	1.004 (0.012)	1.006 (0.004)
Chronic diseases	0.616^***^ (0.028)	0.739^***^ (0.093)	0.519^***^ (0.046)
Smoking	0.847^***^ (0.030)	0.657^***^ (0.105)	0.840^***^ (0.035)
Drinking	1.017 (0.041)	0.861 (0.110)	1.031 (0.040)
Living with parents	0.974 (0.071)	0.974 (0.227)	0.972 (0.069)
Having children	0.582 (0.416)	0.828 (0.962)	0.536 (0.715)
Household size	1.040^***^ (0.007)	1.073^**^ (0.037)	1.025^***^ (0.007)
One of the parents still alive	0.948 (0.033)	0.886 (0.119)	0.936 (0.052)
Pseudo R^2^	0.022	0.030	-
Observations	19,264	19,264	19,264

Note: *** *p* < 0.01, ** *p* < 0.05, * *p* < 0.1. Odds Ratio are reported and robust standard errors are in brackets. These models controlled for the dummy variables of year and province

### Subgroup analysis

We conduct subgroup analysis across five individual traits: gender, area of residence, marriage, working status and whether they live with their parents. For brevity, we only report the results from fixed effects ologit model. The estimated results are shown in Table 4.

**Table 4:** Subgroup analysis in the China family panel studies 2016, 2018,2020

Variables	Subjective wellbeing							
	Female		Male		Urban		Rural	
Informal caregiving	0.829^***^ (0.053)		0.895 (0.091)		0.874^**^ (0.063)		0.869^***^ (0.044)	
Low-frequency of care		0.914 (0.150)		0.903 (0.105)		0.998 (0.142)		0.811 (0.130)
High-frequency of care		0.761^*^ (0.124)		0.882 (0.125)		0.791^*^ (0.106)		0.733^***^ (0.113)
Observations	8,333	8,333	10,931	10,931	10,151	10,151	9,113	9,113
Variables	Married		Unmarried		Working		Not working	
Informal caregiving	0.887^**^ (0.035)		0.925 (0.144)		0.809^***^ (0.072)		0.887 (0.314)	
Low-frequency of care		0.896 (0.103)		0.995 (0.188)		0.824^*^ (0.086)		0.653 (0.603)
High-frequency of care		0.847^*^ (0.087)		0.818^**^ (0.099)		0.788^**^ (0.093)		0.542 (0.242)
Observations	14,417	14,417	4,847	4,847	16,722	16,722	2,542	2,542
Variables	Living with parents				Not living with parents			
Informal caregiving	0.852^*^ (0.072)				0.673^***^ (0.196)			
Low-frequency of care			0.873 (0.086)				0.882 (0.200)	
High-frequency of care			0.821^*^ (0.092)				0.305^***^ (0.352)	
Observations	18,470		18,470		794		794	

Note: *** *p* < 0.01, ** *p* < 0.05, * *p* < 0.1. Odds Ratio are reported and robust standard errors are in brackets. These models controlled for the dummy variables of year and province. The dummy variables of year and province and other variables shown in Table 3 are controlled in all regressions

### Mechanism analysis

Table 5 presents the estimated effects of informal caregiving on the three potential channels. As expected, informal caregiving significantly increased the likelihood of suffering from depression and reduced the wage income, leisure, and social activities of caregivers.

**Table 5:** Mechanism analysis in the China family panel studies 2016, 2018,2020

Variables	Stress channel		Wealth channel		Leisure and social activities channel			
	Depression		Log of wage income		Leisure time		Sleep time	
Informal caregiving	0.262^*^ (0.146)		-0.298^***^ (0.065)		-0.157^*^ (0.092)		-0.037^**^ (0.018)	
Low-frequency of care		0.113 (0.011)		-0.113 (0.074)		-0.070 (0.154)		-0.032 (0.021)
High-frequency of care		0.375^***^ (0.146)		-0.668^***^ (0.095)		-0.336^*^ (0.198)		-0.048^*^ (0.028)
Observations	19,264	19,264	19,264	19,264	19,264	19,264	19,264	19,264

Note: ^***^ *p* < 0.01 ^**^ *p* < 0.05 ^*^ *p* < 0.1. The robust standard errors were reported in brackets and coefficients were reported outside brackets. Ref.=No care provided. The dummy variables of year and province and other variables shown in Table 3 are controlled in all regressions

### Sensitivity analysis

We employ three sets of supplementary analysis to check the robustness of our results. First, we check whether the results were sensitive to the measure of subjective well-being. Panel A of Table 6 reports results in which subjective well-being was measured by happiness. The results indicated that informal caregiving also had a significant negative effect on caregivers’ happiness. Second, considering that the parents of the 16–25-year-old sample are younger and have fewer care needs, we restrict the sample to those aged 25–50 years old. Panel B of Table 6 indicates that informal caregiving significantly reduced the wellbeing, and providing high frequency of care had a greater negative impact on wellbeing, which again verified the robustness of the above regression results. Third, we use the Heckman selection model to address the possible missing data issue, that is, the respondents in the panel data may be missing non-randomly. We first estimated the probability of sample loss using observed individual characteristics and instrumenting with the attrition rate measured at the district and county level. Then, we derived the inverse of the predicted probability (Mill’s ratio) and added it as a covariate to the regression model. Panel C of Table 6 reports results of Heckman selection model. The coefficient of the Mill’s ratio was not statistically significant, indicating that there is no sample selection in the analysis. The coefficients of informal caregiving and different frequency of care are still significantly negative, indicating that the negative effect of informal caregiving on caregivers’ subjective well-being remains robust.

**Table 6:** Sensitivity analysis in the China family panel studies 2016, 2018,2020

Panel A: Subjective wellbeing is measured by happiness		
Variables	Happiness	
Informal caregiving	-0.168^***^ (0.029)	
Low frequency of care		-0.142^***^ (0.032)
High frequency of care		-0.222^***^ (0.044)
Observations	19,264	19,264
Panel B: Sample restricted to 25-50 years old		
Variables	Subjective wellbeing	
Informal caregiving	0.783^**^ (0.097)	
Low frequency of care		0.811^**^ (0.094)
High frequency of care		0.768^**^ (0.108)
Observations	3,258	3,258
Panel C: Heckman selection method		
Variables	Subjective wellbeing	
Informal caregiving	0.857^*^ (0.081)	
Low-frequency of care		0.975^*^ (0.015)
High-frequency of care		0.803^**^ (0.088)
Mill’s ratio	-6.257 (5.736)	-6.296 (5.751)
Observations	19,264	19,264

Note: *** *p* < 0.01, ** *p* < 0.05, * *p* < 0.1. Odds Ratio are reported and robust standard errors are in brackets. These models controlled for the dummy variables of year and province. The dummy variables of year and province and other variables shown in Table 3 are controlled in all regressions

## Discussion

This study uses the 2016, 2018, and 2020 waves of data from China Family Panel Studies (CFPS) to investigate the effect of informal caregiving on caregivers’ subjective wellbeing. Specifically, this study finds statistically significant negative associations between informal caregiving and caregivers’ subjective wellbeing. More importantly, we explore the heterogeneous impacts and mechanisms underlying the effect.

Our findings confirm the first hypothesis that informal caregiving significantly reduces caregivers’ subjective wellbeing. and the negative effect is more pronounced for caregivers who provided high frequency of care. The negative causal impact remained robust after addressing endogeneity using fixed effects models and extended ordered probit regression. Our findings are consistent with studies by Chen et al. [23] and Liu et al. [24], who also conclude that informal caregiving significantly reduced caregivers’ subjective wellbeing in China.

Moreover, our analysis demonstrates that, compared with men, informal caregiving significantly reduced the subjective well-being of female caregivers and the magnitude of the negative effect was greater among women who provided high-frequency of care. This finding is consistent with the study by Heger [38]. One of the arguments for gender differences is that due to the role of social and cultural norms, women are more aware of their role as caregivers. When they feel that they are not doing well enough, they will feel more pressure. In addition, women are more likely to report their own stressful experiences than men. In terms of area of residence, informal caregiving significantly reduced the subjective wellbeing of urban caregivers, and the negative impact on rural caregivers was greater and more significant. The possible explanation is that urban caregivers enjoy obvious advantages in terms of economic development and elderly care facilities, and they can make full use of social care resources to mitigate the caregiving burden.

Our estimates further suggest that the effect of informal care on caregivers’ subjective well-being is different across marriage, working status and whether they live with their parents. Specifically, informal caregiving significantly reduced married caregivers’ subjective wellbeing, but no significant relationship existed between caregiving and unmarried caregivers’ subjective wellbeing. This is mainly due to the inverted pyramid family structure like sandwich generation, which imposes a heavy caring burden on married groups. As for working status, we found that caregiving significantly deteriorated the subjective wellbeing of working group. The reason may be that children with jobs are under work pressure and have the responsibility of caring for their elderly parents, which making them under double pressure and more likely to show negative emotions such as anxiety and depression. In addition, informal caregiving significantly reduced the subjective wellbeing of caregivers who lived with their parents, but the negative impact on caregivers who did not live with their parents was greater and more significant, which may be due to the extra time and transportation costs incurred in providing informal caregiving for caregivers who did not live with their parents.

Regarding the mechanisms underlying the effect, compared with non-caregivers, caregiving increased caregivers’ depression by 0.262 standard deviation. High-frequency caregiving increased the caregivers’ depression by 0.375 standard deviation. Meantime, informal caregiving reduced caregivers’ wage income by 0.298 standard deviation, and high frequency of care reduced wage income by 0.668 standard deviation. This is consistent with Chen et al. (2019) who found that family caregiving will not only bring explicit economic costs to caregivers but also bring hidden economic costs to caregivers. As for the leisure and social activities channel, caregiving reduced caregivers’ time on leisure and sleep by 0.157 and 0.037 standard deviations, respectively. This is due to time constraints caused by informal caregiving.

### Implications for policy, practice, and future research

This study has important implications for policy makers to design interventions to mitigate the negative impact of informal caregiving on caregivers’ subjective wellbeing. In the process of promoting informal care provision, policy makers should consider the tradeoff between caregiving and caregivers’ well-being. As a possible consequence, the loss of caregiver’s wellbeing may reduce the quality of informal care and increase the demand for institutional care. To sustain a pool of sufficient informal caregivers in the future, policies are needed to mitigate the negative effects of informal care. First, policy makers should provide a series of supportive services for caregivers such as respite service, temporary care, and psychological counseling. Second, special attention should be paid to women, living in rural areas, married, working, and living separately from parents, and the policy makers should provide financial assistance for them. Finally, policy makers should introduce programs to improve caregivers’ stress-coping skills and ensure their engagement in leisure and social activities.

Our study suffers from several limitations. First, while the panel data allows us to control for the presence of time invariant unobserved individual heterogeneity, there may be time varying unobservable that may bias our estimated results. Second, we were unable to estimate the long-term effects of informal caregiving on caregivers’ wellbeing. Third, our measure of informal care is self-reported, which may suffer from measurement error.

## Conclusion

This study finds that that informal caregiving significantly reduced caregivers’ subjective well-being and the negative effect is more pronounced for caregivers who provided high frequency of care. The negative causal impact remained robust after addressing endogeneity using fixed effects models and extended ordered probit regression. Subgroup analysis shows that the negative impact of informal caregiving on caregivers’ subjective well-being differed significantly across individual characteristics as well as family characteristics. Informal caregiving and high frequency of care has greater negative impact on their subjective well-being on women, those living in rural areas, married, working, living separately from their parents, and having children. Mechanism analysis further reveals that informal caregiving and different frequency of care have a negative impact on caregivers’ subjective well-being by increasing psychological depression and reducing caregiver’s wage income, leisure, and sleep time.

Our study calls for more research on long-term effect of informal caregiving and developing more sophisticated methods to establish the causal relationship between informal caregiving and caregivers’ subjective wellbeing.

## Data Availability

The data used in this study is from China Family Panel Studies (CFPS), which can be accessed at www.isss.pku.edu.cn.

## Footnotes

1 The BUC estimator combines the LL functions resulting from different cutoff points, leading to a one-step estimator of ${\upbeta }$. The LL function for this estimator is$L{L}^{BUC}\left(b\right)={\sum }_{k-2}^{K}L{L}^{k}\left(b\right)$, where the BUC estimator is the one that maximizes LL function. We call this the BUC estimator because this describes how the estimator is implemented: first, all individual’s observations in the sample are replaced with K−1 copies or clones of itself (“blow up” the sample size); and, then, each clone is dichotomized at a different cutoff point. We then use the entire inflated sample to estimate β by applying the CML estimator. Because the clones of the same individual are not independent of each other, we must compute standard errors that are clustered at the individual level.
